# I’ll Never Forgive You: High Conflict Divorce, Social Network, and Co-Parenting Conflicts

**DOI:** 10.1007/s10826-017-0821-6

**Published:** 2017-06-15

**Authors:** Margreet Visser, Catrin Finkenauer, Kim Schoemaker, Esther Kluwer, Rachel van der Rijken, Justine van Lawick, Hans Bom, J. Clasien de Schipper, Francien Lamers-Winkelman

**Affiliations:** 1 Department of Clinical Child and FamilyStudies, VU University Amsterdam, Van der Boechorststraat 1, 1081 BT Amsterdam, The Netherlands; 2 EMGO Institute for Health and Care Research, Van der Boechorststraat 7, 1081 Amsterdam, The Netherlands; 3 KJTC (Children’s Trauma Center Haarlem), Haarlem, The Netherlands; 4 University Utrecht, Domplein 29, 3512 JE Utrecht, The Netherlands; 5 Viersprong Institute for Studies on Personality Disorders, Postbus 7, 4660 AA Halsteren, The Netherlands; 6 Lorentzhuis (Centre for Family Therapy, Training and Consultation), Van Eedenstraat 16, 2012 EM Haarlem, The Netherlands

**Keywords:** High conflict divorce, Forgiveness, Social network, Co-parenting conflicts, Parental adjustment

## Abstract

The relation between divorce, co-parenting conflicts, and children’s adjustment problems has been well established. An unresolved question for research and clinical interventions, however, is how conflicts between parents are maintained and/or escalate. This cross-sectional research tested the hypothesis that co-parenting conflicts in divorced couples are associated with perceived social network disapproval and that this relation is mediated by parents’ tendency to forgive each other. In Study 1, a convenience sample of 136 divorced parents recruited via online forums, we showed that perceived social network disapproval was indeed positively related to co-parenting conflicts and that parents’ tendency to forgive the other parent—albeit partly—explained this relationship. Strength of our research is that in Study 2, 110 parents referred to children’s mental health care because the wellbeing of the children was severely compromised by the severity of the conflicts between parents, we replicated these results. In both studies perceived social network disapproval and co-parenting conflicts were positively related and this link was mediated by forgiveness: perceived social network disapproval was negatively related to forgiveness, which in turn was negatively related to more parental conflicts.

## Introduction

Living in divorced families is common (Spruijt and Kormos 2010) and may be harmful for children (Amato 2001). In the Netherlands, approximately 70,000 children experience parental divorce every year (Spruijt and Kormos 2010). The most devastating effect of divorce for children’s adjustment and well-being is being exposed to parental conflict (Amato 2001; Kelly and Emery 2003). Consequently, one of the most challenging tasks for parents after divorce is to establish a high quality co-parenting relationship. This is crucial, not only for parental adjustment and wellbeing (Katz and Woodin 2002), but also because co-parenting quality is essential to ensure children’s healthy and smooth adaptation to divorce (Amato 2005; Bronstein et al. 1993; Nunes-Costa et al. 2009; Whiteside 1998) and prevent developmental decrements in the long-run (Cabrera et al. 2012; Levine and Painter 1998; Prevoo and Ter Weel 2014).

An important question for research then is to explain how conflict between divorced parents is maintained and/or how it escalates. Although research has examined risk factors for co-parenting conflicts (see for an overview, Bonach 2005), and increased our knowledge about conflict escalation (Coleman et al. 2012), one aspect that has received little attention in empirical research is the role of the social network, including friends, family, and even lawyers (Milardo et al. 2014). This oversight is surprising, given that it is generally recognized that the success and failure of relationships does not only depend on the individual partners but also on their social networks, both in intact relationships (Kennedy et al. 2015) and post-divorce relationships (McDermott et al. 2013). As an example, it has been found that social network approval is an important protective factor for the quality of romantic relationships (Le et al. 2010). Also, social network support was found to be an important protective factor for parents’ individual adjustment after divorce (Albeck and Kaydar 2002; Kramrei et al. 2007).

To explain how social network approval or disapproval may influence the level of co-parenting conflicts, we extend findings on the so-called third-party forgiveness effect (Green et al. 2008) to divorced families. In these families, social network members, like family and friends, can be regarded as third parties in transgressions made between parents. Research has shown that third parties are generally less forgiving than first parties (Green et al. 2008), for example, because they may benefit less from repairing the relationship (Green et al. 2008). Applying these findings to divorced parents, we suggest that friends, family, and important others are reluctant to forgive transgressions made by the ex-partner, in the past and in the present. Consequently, the social network is likely to bring up (past) transgressions of the ex-partner and speak negatively about the ex-partner, which is perceived by parents as disapproval of the ex-partner.

Co-parenting can be conceptualized as the parental relationship in the planning and execution of a joint parental plan for the children. It can be defined as “the joint and reciprocal involvement of both parents in the education, background and decision-making about their children’s lives. Cooperative parents prioritize their children’s well-being, while creating and maintaining a constructive relationship, with new, more flexible boundaries between one another” (Nunes-Costa et al. 2009, p. 388). Furthermore, it is important that parents support each other’s educational decisions (Maccoby et al. 1990) and parental efforts (McHale et al. 2004; Whiteside and Becker 2000). In support of these suggestions, Whiteside and Becker (2000) found that high levels of positive supportive co-parenting are negatively associated with conflicted co-parenting.

A majority of divorced parents succeeds in remaining supportive of one another and develop a cooperative co-parenting style (Whiteside 1998; Whiteside and Becker 2000). They communicate frequently, although they often have different opinions when parental and educational decisions concerning the children need to be made (e.g., Maccoby et al. 1990). However, approximately one third of divorced parents have high levels of ongoing hostility and tension (Whiteside 1998). The combination of differing opinions and high levels of ongoing hostility and tension between parents may result in unresolved conflict and contribute to the escalation of co-parenting conflicts (Bonach 2005; Coleman et al. 2012).

Ample evidence shows that social network support is important for the individual well-being of parents (Pinquart and Sörensen 2000). Furthermore, research has shown that network relationships (being part of a group), more than specific relationships (one-on-one contact), promote positive post-divorce adjustment, including adaptive coping, mental wellbeing, and life satisfaction (Kramrei et al. 2007). This highlights that being part of a supportive social network is particularly important for healthy adjustment after divorce. Social networks provide divorced parents with a feeling of belongingness and offer emotional support, for example, by approving of the relationship breakup and making negative statements about the ex-partner (Sprecher and Felmlee 2000). Thereby, social networks may help the individual ex-partners to feel better by increasing their sense of belonging as well as by decreasing feelings of uncertainty about ending the romantic relationship (Eaton and Sanders 2012). Despite its beneficial effect for individual post-divorce adjustment, however, such social network support might at the same time have an escalating effect on conflict with the ex-partner. When network members express themselves negatively about the ex-partner as an act of support, they also fuel their divorced friend’s or family member’s negative thoughts, feelings, and behaviors regarding the ex-partner (Lickel et al. 2006).

Forgiveness is an interpersonal process (for a review see Karremans and Van Lange 2008), which serves to maintain the relationship after a transgression has been committed, and to rebuild the quality the relationship had before the transgression. In relationships, including post-divorce relationships, partners intentionally or unintentionally hurt or offend each other. They may lie about extramarital affairs, are emotionally absent, disclose secrets, break promises, or gossip about each other with their friends. To effectively deal with these inevitable transgressions and prevent conflict, relationship partners need to forgive each other (Karremans and Van Lange 2008). Not surprisingly, empirical research consistently finds that forgiveness has profound consequences for the forgiving individual, such as beneficial effects for psychological and physical health, greater life satisfaction, and lower levels of psychological distress (Karremans et al. 2003; Lawler et al. 2005; McCullough et al. 2001). Forgiveness also plays a crucial role in relationships. For example, it is associated with less conflict and greater relationship quality in romantic relationships (Paleari et al. 2005) and more cohesion in families (Maio et al. 2008). Last but not least, forgiveness not only affects individuals and relationships, but also their social network (Green et al. 2014). People close to the victim of a transgression, so-called third parties (Green et al. 2008), who are not directly involved in the transgression, may feel that they are in a position to grant or withhold forgiveness themselves, and/or influence the forgiveness process of the victim.

Research shows that third parties are generally less forgiving than victims themselves and offers several explanations for this third party forgiveness effect (for a review see Green et al. 2014). For example, family, friends, or other important network members may be afraid to jeopardize their close relationship with the victim by being forgiving toward the perpetrator. Furthermore, given that they have less information about the perpetrator than the victim does, social network members may blame the perpetrator more for what happened, and make more negative, internal, and stable attributions about the perpetrator. Finally, research indicates that third parties are less likely to believe apologies and see less profit in reconciliation than do victims themselves (Cheung and Olson 2013; Eaton and Sanders 2012; Green et al. 2008, 2014).

Interpersonal transgressions are important stressors before, during, and after divorce, which may contribute to the maintenance and escalation of co-parenting conflict (Bonach 2005). Research on clinical interventions for divorcing couples suggests that, in these couples, forgiving the other parent is crucial, not only because forgiveness is negatively related to conflicts, but also because it is positively related to the quality of the co-parenting relationship (Reilly 2014; Rye et al. 2012). Furthermore, forgiveness is one of the strongest predictors of the quality of co-parenting over time (Bonach 2005; Bonach and Sales 2002).

It is possible that parental education, the length of the relationship, and time since separation are linked to the key variables in our research (Yárnoz Yaben 2009). Also, although both men and women tend to increase mobilization of social network support in times of greater distress (Fincham et al. 2007), gender differences may affect the hypothesized processes, especially because men were found to be more forgiving than women (Sidelinger et al. 2009).

The aim of the present research was to examine the indirect relation between perceived social network disapproval and co-parenting conflicts via forgiveness in the divorce context. Our first hypothesis was that among divorced parents the level of perceived social network disapproval is positively related to co-parenting conflicts. Our second hypothesis was that parental forgiveness is negatively related to more co-parenting conflicts. Our third hypothesis was that the association between perceived network disapproval and co-parenting conflicts is mediated by parental forgiveness of the other parent/ex-partner. We tested our predictions, first, in a convenience sample of divorced parents recruited via online forums, and, second, in a clinical sample of parents involved in high-conflict divorces who were referred to treatment because of the imminent threat their conflicts posed to the psychosocial wellbeing of their children. To rule out possible confounding influence of parental gender and education, the length of the relationship, and the time since separation, we examined their influence in both studies.

## Study 1

In Study 1, we sought to provide evidence for our mediational model that perceiving social network disapproval of the other parent is associated with greater conflict between divorced parents. We also expected that divorced parents’ forgiveness toward the ex-partner would mediate the association between network disapproval and co-parenting conflict.

## Method

### Participants

Participants were 136 divorced parents (mean age 44.5 years, SD = 5.8, range 27–58 years). None of the participants were each other’s ex-partner as far as we know. Ninety-six percent was Dutch. On average, they had two children with their ex-partner (SD = 0.7, range = 1–4). The oldest child had a mean age of 13.8 years (SD = 5.0, range 4–25 years). Forty-nine percent of the parents had a new relationship (*n* = 66), and only 3% had children in their new relationship (*n* = 4). Fifty-two percent sought professional help (e.g., therapy) to adjust to the divorce (*n* = 70).

### Procedure

We recruited divorced parents by posting announcements with a link to the online questionnaire on a variation of Dutch general and divorce-related websites, forums, and social media (e.g., Facebook, www.villapinedo.nl, www.nieuwstap.nl, www.singlesite.nl, Psychologie Magazine, Marktplaats), by sending e-mails with a link to the questionnaire to family, friends, and acquaintances, and by posting announcements in online newsletters of divorce mediation agencies. Parents filled in an online questionnaire about themselves, their children, their ex-partner, and their current relationship with the other parent. Only demographic characteristics and the measures central to our research questions will be described below. To avoid possible confounding influence of complex, high-conflict divorce cases in this study, we excluded parents with ongoing legal procedures with the other parent (*n* = 26). All participants gave informed consent before completing the questionnaires. As a reward for participating, they received a gift-voucher of 7.50 Euro for an online web-shop.

### Measures

#### Demographic information, family and divorce measures

To collect socio-demographic information about the participants, they answered questions about their age, gender, level of education, and ethnicity. Additionally, several questions assessed information about family and divorce characteristics including number of children, time since divorce, seeking of help to adjust to divorce, duration of marriage/legal cohabitation, and new relationship. Gender, level of education, time since separation, and duration of marriage/legal cohabitation were used as control variables.

#### Co-parenting conflicts

To assess co-parenting conflicts, we used the 7-item co-parenting subscale of The Psychological Adjustment to Separation Test (PAST; Sweeper and Halford 2006). The scale was translated into Dutch and showed good psychometric properties (De Smet 2013). Example items are: “When I speak to my former partner we usually fight over the child/children” “My former partner and I avoid speaking to one another”. Items were rated on a 5-point scale (1 = *strongly disagree*, 5 = *strongly agree*). Mean scores were calculated such that a higher score indicated more co-parenting conflicts (Cronbach’s alpha = .89).

#### Perceived network disapproval

To assess parents’ perception of the extent to which their social network disapproves of the ex-partner, we first asked each parent to make a list of people who are involved in and concerned by the divorce (e.g., lawyers, parents(-in-law), friends, new partners). Subsequently, participants completed four questions assessing their perception of network partners’ overall reactions to the divorce, including questions concerning their (dis)approval (e.g., “in general, my network supports rapprochement and compromise with my ex-partner (reversed)” (cf Lehmiller and Agnew 2007). Items were rated on a 5-point scale (1 = *not at all*; 5 = *very much*). Mean scales were calculated with a higher score indicating higher levels of perceived social network disapproval (Cronbach’s alpha = .65).

#### Forgiveness

To assess feelings of forgiveness, we used a twelve-item Dutch translation of the Transgression Related Interpersonal Motivations Inventory (McCullough 2013), rated on a 5-point scale (1 = *strongly disagree* to 5 = *strongly agree*). Parents rated their feelings of forgiveness toward the ex-partner (e.g., “I keep as much distance as possible from my ex-partner.” (reversed); “I want to see my ex-partner hurt and miserable.” (reversed); “Although my ex-partner hurt me, I am putting the hurts aside so we can resume our contact.”). Mean scale was calculated such that a higher score indicated a higher level of forgiveness (Cronbach’s alpha = .91).

### Data Analyses

Descriptive analyses were conducted to examine family and divorce, and social network characteristics, possible gender differences, and zero-order correlations among all study related variables. Second, we used ordinary least squares path analyses to conduct simple mediation analyses, to test whether forgiveness explained—albeit partly—the relation between perceived social network disapproval and co-parenting conflicts. All analyses were conducted in IBM SPSS Statistics version 21 (SPSS 2012), in which we used macro PROCESS for mediation analyses, model 4 (Hayes 2013). We controlled for parental relationship length, gender, time since separation, and educational level to rule out alternative hypotheses and the influence of confounding variables.

## Results

Seventy-two percent of the participants were mothers. The educational level was moderate (41% completed secondary vocational education) to high (57% completed higher vocational education and university). Participants had had a relationship with their ex-partner before divorce for 16.1 years (SD = 7.2; range 2–35 years), and had been separated for 4.7 years (SD = 4.0; range 0–16 years). Participants reported a mean of five persons (SD = 3.0) in their social network (range 0–10), 34% own family, 1% family of the other parent, 44% own friends, 0% friends of the other parent, 6% psychological counselors, 3% legal workers, 6% new partner, 5% other not specified, and 4% reported to have nobody.

Independent-samples t-tests were conducted to compare the study variables for fathers and mothers. Results indicated that perceived social network disapproval, forgiveness, and co-parenting conflicts did not differ significantly across gender, *t*(134)  ≤  1.361, *p* *≥*  .179, *d * ≤ .02334.

Means, standard deviations, for fathers and mothers, and bivariate correlations among study related variables, are presented in Table 1. Consistent with the first hypothesis, higher levels of perceived network disapproval were significantly related to more co-parenting conflicts, *r*(134) = .611, *p * <  .001, and to lower levels of forgiveness, *r*(136) = −.521, *p*  <  .001. Also, consistent with our second hypothesis, lower levels of forgiveness were significantly related to more co-parenting conflicts (*r*(134) = −.536, *p * <  .001).

**Table 1:** Descriptives and zero-order correlates of all study variables study 1

Study 1 *n* = 136						
Variable	Mean		SD		1.	2.
	Male	Female	Male	Female		
1. Network disapproval	2.95		.91			
	3.07	2.91	.88	.92		
2. Co-parenting conflicts	2.36		1.04		.611**	
	2.58	2.28	1.17	.98		
3. Forgiveness	3.56		.89		−.521**	−.536**
	3.49	3.58	.90	.90		

*p *<* .05; **p *<* .01

Consistent with our mediation hypothesis, simple mediation analyses using ordinary least squares path analysis yielded that perceived social network disapproval indirectly influenced the amount of co-parenting conflicts through its effect on forgiveness. As presented in Table 2, parents who perceived more disapproval in their social network were less likely to forgive the other parent (*b* = −.512, *p* *<* .001), and when parents were less likely to forgive the other parent, they reported more co-parenting conflicts (*b* *=* −.347, *p* *<* .001). We calculated bias-corrected bootstrap confidence intervals estimated based on 5000 bootstrapped samples and a 95% confidence interval. The indirect effect (ab) of perceived network disapproval through forgiveness on co-parenting conflicts, did not include zero (for more details see Table 2), which indicates that the effect is significant.

**Table 2:** Forgiveness (F) as a mediator between perceived social network disapproval (ND) and co-parenting conflicts (CC) in divorced families (*n* = 131)

Model	ab	95% CI		*k* ^2^	*c* (*p*)	*c*′(*p*)
		*LL*	*UP*			
ND → F → CC	.179	0.0671	0.3063	.1684	.700 (.000)	.523 (.000)
ND → F → CC (with covariates)	.161	0.0530	0.2909	–	.676 (.000)	.515 (.000)

*Note*: Unstandardized regression weights are presented. *k* ^2^ represents kappa, an effect size measure for indirect effects. *c* represents the direct effect of perceived social network disapproval on co-parenting conflicts. *c’* represents the direct effect of perceived social network disapproval on co-parenting conflicts, controlling for forgiveness. Covariates are educational level, relation length, time since separation and gender

Also, the indirect effect (ab), controlling for the effect of parental educational level (*b* *=* .011, *se* *=* .043, *p* *=* .803), length of parental relationship (*b* *=* −.000, *se* *=* .001, *p* *=* .782), time since separation (*b* *=* −.037, *se* *=* .018, *p* *=* .047), and gender (*b* = −.002, *se* *=* .151, *p* = .989), of perceived network disapproval through forgiveness on co-parenting conflicts, did not include zero (for more details see Table 2), which indicates that the effect remained significant when controlling for possible confounders. As can be seen in Table 2, perceived social network disapproval remained a significant direct predictor of co-parenting conflict after controlling for the level of forgiveness, which indicates that other factors, at least, mediate the relation between perceived network disapproval and co-parenting conflict.

## Discussion

Extending previous research on social network disapproval and forgiveness to co-parenting conflicts between divorced parents, we predicted that forgiveness mediates the link between perceptions of network disapproval and conflict. The findings from Study 1 support our hypotheses. They provide initial evidence for the relation between perceptions of network disapproval and co-parenting conflict and document that forgiveness is a critical mechanism of this effect. Specifically, we predicted, and found, an indirect relation between perceived social network disapproval and co-parenting conflicts through parents’ tendency to forgive the other parent, but the direct effect also remained.

Although these findings are encouraging, Study 1 included a convenience sample of divorced parents recruited via online forums, thereby reducing the generalizability of our findings. This is especially important, given that self-selection may have biased our sample. For example, it is possible that only well-adjusted divorced parents participated. Therefore, it remains unclear whether our findings can be replicated among divorced couples with high conflict levels. Given the devastating effects of co-parenting conflicts on children’s post-divorce adjustment and well-being (Amato 2001; Johnston 1994; Kelly and Emery 2003), and the fact that high conflict parents often underestimate the effects of their conflicts on children (Anderson et al. 2010), a replication of our findings in a high conflict sample of parents was deemed necessary.

## Study 2

Our second study was guided by two central goals. First, it aimed to include divorced parents with high conflict levels. Second, we also sought to include more men to examine the robustness of our findings on gender differences in Study 1 (28% fathers). This is especially important because fathers’ features and behavior are related with children’s normal and abnormal development (Cassano et al. 2006), but they are underrepresented in pediatric research and in therapeutic treatment of children’s mental health (Phares et al. 2005).

## Method

### Participants

Participants were 110 parents (mean age 42.6, SD = 5.6, range 26–60 years) who were referred for intervention at several family treatment centers in the Netherlands, because the wellbeing of their children was threatened by parents’ long-lasting conflicts, aggression, and anger surrounding parental decisions. Men had a mean age of 43.3 (SD = 6.2, range 27–60), and women had a mean age of 42.0 (SD = 5.0, range 26–56). Ninety-six percent of the sample was native Dutch or Belgian. From 32 families only one parent participated, and from 39 families both parents participated. The 110 parents had 127 children, with a mean of 1.79 children (SD = 0.7) and the mean age of the oldest child was 10.9 years (SD = 3.6). Seventy-four percent of the parents had a new relationship (*n* = 72), and 27% had children in their new relationship (*n* = 19). One hundred percent had sought professional help to adjust to the divorce. Of the 173 parents who were invited to participate, 110 agreed, resulting in a response rate of 64%.

### Procedure

Parents were recruited from ten outpatient health care institutions in different urban and rural regions of the Netherlands and Belgium. The study was part of a larger ongoing study. All parents were referred by judges, Youth Care Agencies (in Dutch: Bureau Jeugdzorg), or a physician, because the wellbeing of the children was severely compromised by the severity of the conflicts between the parents. After the referral, parents enrolled voluntarily in the intervention No Kids in the Middle (Van Lawick and Visser 2015).

Parents were invited for clinical intake as soon as they had both signed up for the intervention separately. Together with the written invitation, parents received information about the research project entitled ‘Parenting in the Aftermath of Divorce and No Kids in the Middle: an ongoing study among divorced families’. During the first clinical intake, all questions parents had about the research were answered and the consent form was signed. Subsequently, the clinician informed the researcher and the researcher sent an email to parents with their personal code and a link to the online questionnaire. All questionnaires were programmed in Qualtrics, an online survey software program. Parents were asked to complete the online questionnaire before the second clinical intake or at least before the start of the intervention.

### Measures

In Study 2, we used the same measures as in Study 1 to assess demographic information and family and divorce measures, control variables, co-parenting conflicts (Sweeper and Halford 2006) (*α* = .75), perceived network disapproval (Lehmiller and Agnew 2007) (*α* = .62), and forgiveness (McCullough 2013) (*α* = .91).

### Data Analyses

Like in Study 1, descriptive analyses were conducted to examine family, social network, and divorce characteristics, and possible gender differences. Second, to examine whether we successfully included a high-conflict divorce sample, we conducted an independent t-test to examine whether high conflict divorced parents in Study 2 showed more co-parenting conflicts than the divorced parents in Study 1. Third, we examined the same mediational model as in Study 1. Because data provided by two partners in a couple are not independent, even though they are ex-partners, we analyzed the data in Study 2 using hierarchical linear modeling (Raudenbush and Bryk 2002). In our analyses, data from the two ex-partners were nested within couple in a two-level hierarchical linear model. Because none of our effects were moderated by participant sex and because we had one lesbian couple, dyads were treated as indistinguishable (Kenny et al. 2006). We represented intercept terms as random effects and represented slope terms as fixed effects as recommended for (ex)-couples’ data (Kenny et al. 2002). Again, we controlled for parental relationship length, gender, educational level, and time since separation to rule out alternative hypotheses and the influences of confounding variables.

## Results

Forty-six percent of our sample was male, so we succeeded to include more men in Study 2 than in Study 1. The educational level was moderate (46%, secondary vocational education) to high (53%, higher vocational education and university), and only 1% had a low level of education (lower vocational education). On average, parents had had a relationship with their ex-partner for 12.0 years (SD = 6.3; range 0–26), and had been separated for 4.6 years (SD = 2.9; range 0–12). Participants reported a mean of six persons (SD = 2.8) in their social network (range 0–10), 31% own family, 1% family of the other parent, 34% own friends, 0% friends of the other parent, 8% psychological counselors, 7% legal workers, 6% new partner, 12% other not specified, and 3% reported to have nobody.

To explore possible gender differences, we conducted multilevel analyses, controlling for interdependence between ex-partners, to compare the study variables for fathers and mothers. The results indicated that perceived social network disapproval varied as a function of gender, *B* *=* .11, *t*(58.59) = 2.19, *p* *=* .033, 95% CI = [0.022, 0.4967]. Women reported higher levels of perceived social network disapproval than men (for details see Table 3). We found no gender differences for forgiveness *B* *=* −.12, *t*(65.68) = −.082, *p* *=* .413, 95% CI = [−0.412, 0.171]. Co-parenting conflicts did differ significantly across gender in the high conflict divorced group, *B* *=* −.26, *t*(57.34) = −2.55, *p* *=* .013, 95% CI = [−0.472, −0.057]; men reported higher levels of co-parenting conflict than women (for more information see Table 3).

**Table 3:** Descriptives and zero-order correlates of all study variables Study 2

Study 2 *n* = 110						
Variable	Mean		SD		1.	2.
	Male	Female	Male	Female		
1. Network disapproval	3.31		.75			
	3.19	3.40	.78	.73		
2. Co-parenting conflicts	3.34		.72		.262**	
	3.46	3.23	.75	.68		
3. Forgiveness	3.23		.79		−.301**	−.408**
	3.28	3.18	.73	.84		

*p *<* .05; **p *<* .01

Also, an independent-samples t-test examined hypothesized group differences for co-parenting conflicts. As expected, the sample of divorced parents in Study 2 scored significantly higher on co-parenting conflicts (*M* = 3.34, SD = .72) than the sample in Study 1 (*M* = 2.36, SD = 1.04), *t*(235) = 8.666, *p* < .001, *d* = 1.1297 (this is a large effect size). So, our recruitment strategy successfully resulted in the inclusion of parents involved in high-conflict divorces.

The pattern of zero-order correlations in Study 2 (see Table 3 for more details) closely replicated the one observed in Study 1. Again, higher levels of perceived social network disapproval were significantly related to more co-parenting conflicts (*r*(110) = .262, *p* = .006), and to lower levels of forgiveness (*r*(110) = −.301, *p* = .001). Also, lower levels of forgiveness were significantly related to more co-parenting conflicts (*r*(110) = −.408, *p* < .001).

To explore whether ex-partners’ ratings were related, we examined the intra-class correlations within ex-couples. We found that within the 39 ex-couples, partners’ reports on perceived social network disapproval were significantly correlated ICC = .328, *z* = 2.05, *p* = .020, as were their reports on co-parenting conflict ICC = .448, *z* = 2.80, *p* = .023, whereas their forgiveness was unrelated, ICC = .062, *z* = 0.39, *p* = .348. For none of the variables did we find significant differences between participants in ex-couples and single participants.

To test the hypothesized link between perceived social network polarization and co-parenting conflict, we performed multilevel regression analyses, regressing co-parenting conflict onto perceived social network disapproval. As hypothesized, perceived social network disapproval was positively associated with co-parenting conflict, *B* *=* .18, *t*(107.909) = 2.14, *p* *=* .035. To test whether social network disapproval was associated with forgiveness, we regressed forgiveness on social disapproval. Perceived social network disapproval was negatively associated with forgiveness, *B* *=* −.32, *t*(102.211) = −3.27, *p* *<* .001. To ensure that the results were valid above and beyond educational level, gender, relationship duration and time since separation, we conducted all analyses controlling for these variables. All results remained significant, indicating that perceived social network disapproval reliably accounted for unique variance beyond these control variables, *B* *=* .22, *t*(101.97) = 2.56, *p* *=* .012 in co-parenting conflict and, *B* *=* −.32, *t*(96.82) = −3.13, *p* *=* .002 in forgiveness, respectively.

Furthermore, we assessed whether forgiveness mediated the link between social network disapproval and co-parenting conflict. To test for mediation, because our model included no random slope, we used the Monte Carlo Method for Assessing Mediation (MCMAM: Selig and Preacher 2008). This method was used to generate a 95% CI for the indirect effect with 20,000 resamples. Significant mediation is indicated when the CI does *not* include zero. Regarding the mediation effect, the analyses revealed that forgiveness mediated the effect of perceived social network disapproval on co-parenting conflict (indirect effect: 95% CI = [0.026, 0.177]; direct effect: *B* *=* .11, *p* *=* .191). Taken together, these results suggest that when parents involved in high-conflict perceive their social network to be disapproving of the ex-partner, they are less forgiving and this is related to more co-parenting conflict.

## Discussion

The results of Study 1 were consistently replicated in Study 2. Among parents involved in high-conflict divorces, we found a positive relation between perceived social network disapproval and the number of co-parenting conflicts. Furthermore, results confirmed our hypothesis that forgiveness between ex-partners plays a crucial role in explaining this association. So, the results provide empirical support for the indirect relation between perceived social network disapproval and co-parenting conflicts through parents’ tendency to forgive the other parent in a group of high-conflict parents. By adopting a different recruitment procedure, we succeeded not only in including a high-conflict divorce sample, but also in including more fathers than in Study 1. Additionally, all effects were significant when we ruled out possible statistical interdependence among ex-partners by conducting multilevel regression analyses. All three aspects speak to the robustness of our results.

The results of Study 2 also yielded a number of new insights. First, they revealed that ex-partners showed agreement in their evaluation of co-parenting conflict and perceived social network disapproval, but not their forgiveness. Second, they revealed gender differences in the report of co-parenting conflict and social network disapproval, but not for forgiveness.

The findings regarding the intraclass correlations may be attributable to the fact that co-parenting conflict and perceived social network disapproval happen between people, while forgiveness is an *intra*personal process with *inter*personal consequences. Specifically, to forgive their ex-partner, people have to consciously and actively seek to overcome their negative thoughts, feelings, and behavioral tendencies toward a transgressor to regain a more positive stance, despite the perpetrator’s hurtful actions (McCullough et al. 1998; Worthington 2001). This transformation needs to take place intrapersonally (Worthington 2001), before it can be translated into behavior toward the perpetrator (McCullough et al. 1998). Consequently, there may not be agreement in forgiveness between ex-partners in high-conflict divorce couples. It is possible that there is more agreement when divorced parents manifest behavior interpersonally so that the ex-partners can become cognizant of a positive change in their attitudes towards them (McCullough et al. 1998). A core feature of interpersonal forgiveness is that it is approach-oriented, indicating a willingness to re-engage with perpetrators. For example, McCullough (2008) argues that reconciliation may be a behavioral proxy for interpersonal forgiveness. Future research examining the interpersonal manifestations of forgiveness among high-conflict couples may be particularly promising.

Co-parenting conflict mostly takes place in the presence of the ex-partner, and although ex-partners may not agree on the severity or intensity of the conflict, they do agree on the fact that conflicts take place (Halford and Sweeper 2013). Because parents share responsibility and care for their child(ren) (McHale et al. 2012), they need to interact with each other for co-parenting conflict to occur. This interaction, in turn, may facilitate the accurate detection of conflict. Similarly, social network disapproval can be assumed to be felt by both partners in that their networks need to separate once the divorce has taken place (Sprecher and Felmlee 2000). We will address the mean differences across gender in the general discussion.

## General Discussion

The findings of the two studies presented here shed light on one underlying mechanism that can account for why in many divorced couples co-parenting conflicts are maintained or even escalate. The results showed that parents who perceive more disapproval toward the other parent in their social network after a divorce have more co-parenting conflicts. In addition, the willingness of parents to forgive the other parent’s transgressions explained, at least in part, the link between perceived network disapproval and co-parenting conflicts. Speaking to the robustness of these results, we found the hypothesized mediation across two studies, involving a convenience sample of divorced parents and a sample of high conflict divorced parents whose children were clinically referred for intervention because their wellbeing was severely compromised by the severity of parental conflicts. These findings are in line with a growing body of research demonstrating the importance of the broader social network on relationship processes between (ex) partners (Agnew 2014; Crowley and Faw 2014; Hogerbrugge et al. 2013).

Consistent with our first hypothesis in both studies, we found that divorced parents who perceived more disapproval in their social network had more co-parenting conflicts. Extending previous work on the importance of social network influences on relationship quality in ongoing relationships (Lehmiller and Ioerger 2014), the current research demonstrated that the perception of a negative attitude toward an ex-partner is linked to more parental conflict. Our findings are compatible with the suggestion that ex-partners mobilize social and emotional support to justify the divorce (Sprecher and Felmlee 2000), which may help the individual ex-partners to increase their sense of belonging and decrease feelings of uncertainty (Eaton and Sanders 2012). Despite its beneficial effect for individuals’ post-divorce adjustment (Kramrei et al. 2007), our findings suggest that such perceptions of social network approval of the divorce may be perceived as social network *dis*approval of the other parent and are positively related to conflicts in the co-parenting relationship. Our studies did not allow us to test these processes, because they were correlational and did not include items tapping ex-partners’ strategies to mobilize support (Crowley and Faw 2014). In light of the important implications such insights may have for interventions, longitudinal research on these strategies and the interplay of approval of the divorce and disapproval of the co-parenting relationship would be particularly promising. Another future direction for research may be the actual involvement of social network members to answer the question whether parents’ *perceived* social network disapproval corresponds to parents’ *received* disapproval, and second, whether received disapproval is related to the co-parenting relationship. In a review, Haber et al. (2007) showed that perceived social support is related to relationship quality, but received social support is not.

In line with previous research, we found support for our second hypothesis, that the level of forgiveness is positively related to the quality of the co-parenting relationship among divorced parents (Bonach 2005; Bonach and Sales 2002; Reilly 2014; Rye et al. 2012). These results suggest that parents who are more likely to forgive each other’s transgressions made in the far or recent past, may be more capable to prioritize their children’s well-being and share parenting responsibilities in a mutual supportive and cooperative way (Maccoby et al. 1990; Nunes-Costa et al. 2009). Underlining the important implications these findings have for interventions, a preliminary study by Reilly (2014) in a small sample of high-conflict divorce cases (*n* = 32) provided initial evidence that a psycho-educational intervention focusing on forgiveness (Worthington and Scherer 2004) can promote forgiveness and co-operative co-parenting. More research is needed to examine the role of forgiveness in intervention programs for high-conflict divorces.

Also, we confirmed the hypothesized mediation model in both studies. The results suggest that if parents perceive that friends, family members, and important others are blaming the ex-partner for transgressions and are speaking negatively about the ex-partner, it is harder for parents to forgive the other parent, which seems to be one important relational mechanism in the explanation of the maintenance and escalation of conflicts between divorced parents. While our studies shed light on one potential mechanism underlying the link between perceived social network disapproval and co-parenting conflicts, other mechanisms seem possible. For example, parents who perceive more network disapproval may interpret this disapproval as emotional support for their feelings regarding old marital conflicts (Cabrera et al. 2009), or as support for child custody disputes (Sbarra and Emery 2008).

In contrast to Study 1, in Study 2 we found gender differences in the report of co-parenting conflict and social network disapproval, but not for forgiveness. Regarding these mean differences across gender, we believe that the mean differences for social network disapproval need to be replicated. In fact, in Study 1 we did not find significant differences, whereas in Study 2 mothers reported greater social network disapproval than fathers. If future studies were to replicate the latter findings, it may indicate that mothers are more sensitive to others’ judgments and evaluations than fathers, given their greater focus on others and forming connections (Helgeson 1994). Regarding co-parenting conflict, in both studies fathers reported higher levels of co-parenting conflict, albeit significantly only in Study 2. Other studies too found that co-parenting conflict was higher among fathers (Halford and Sweeper 2013), and many fathers report frustration and conflict in their relationships with their child’s mother (Martinson and Nightingale 2008). Possibly this is attributable to the fact that fathers are more often the non-custodial parent, but research would need to examine this suggestion.

### Strengths, Limitations, and Future Research Directions

It is important to note several strengths and a limitation of the present work. One limitation of the present research is the cross-sectional nature of both studies. Nevertheless, the direction of the proposed associations is consistent with longitudinal studies showing that forgiveness predicts conflict resolution (e.g., Fincham et al. 2007). Although plausible, other directional effects can be proposed. To illustrate, DiDonato et al. (2015) manipulated relationship partners’ forgiveness and found that it predicted how social network partners perceived the relationship of the forgiving individual with the perpetrator. Specifically, more forgiveness was associated with greater perceived commitment, satisfaction, and warmth. These results not only emphasize the need for more experimental and prospective studies investigating the proposed links, but also point to the possibility that parental forgiveness, co-parenting conflicts, and perceived social network (dis-)approval may reinforce each other in a cyclic model.

One important strength is the robustness of the results, which replicated across a convenience sample of divorced parents recruited via online forums and a clinical sample of high-conflict divorced parents. A second strength is the broader relational perspective we took in this research. Till now, research mostly focused on the effects of social support and approval of family and friends on individual parental adjustment after divorce (Kramrei et al. 2007), and on social network influence on partners’ decision to divorce (Hogerbrugge et al. 2013). Our study showed that social network (dis)approval also affects the post-divorce relationship between ex-partners. This is important as more and more divorced parents maintain a co-parenting relationships and (un)forgiveness is especially impactful when divorced parents have frequent contact (Kluwer 2016). Third, in the clinical sample, we were able to include 46% fathers, allowing us to examine gender differences and to exclude their confounding influence in the proposed links. Although fathers’ characteristics and behavior are associated with children’s normal and abnormal development, fathers are underrepresented in child psychopathology research (Cassano et al. 2006), as well as in pediatric research and in therapeutic treatment of children’s mental health (Phares et al. 2005).
